# Low affinity histamine uptake into neonatal rat astrocytes does not involve OCT

**DOI:** 10.1186/2050-6511-13-S1-A32

**Published:** 2012-09-17

**Authors:** Vesna Terbuc, Maša Novak, Katja Perdan-Pirkmajer, Sergej Pirkmajer, Mojca Kržan

**Affiliations:** 1 Department of Pharmacology and Experimental Toxicology Faculty of Medicine, University of Ljubljana, 1000 Ljubljana, Slovenia; 2 Institute of Pathophysiology, Faculty of Medicine, University of Ljubljana, 1000 Ljubljana, Slovenia

## Background

Histamine is a double-protonated molecule with corresponding pK_a_ values of 5.8 and 9.4. Therefore, at physiological pH, histamine exists as an equilibrium mixture of tautomeric cations: the monocation making up 96%, the dication only 3% and the rest being nonprotonated histamine. As a protonated molecule histamine most probably uses a carrier protein in order to cross the cell membrane. In the present work we wanted to determine the kinetic properties of histamine uptake and the influence of other biogenic amines on its transport.

## Methods

We performed histamine uptake assays in the model system of cultured neonatal rat astrocytes. The mRNA expression of organic cation transporters (OCTs) was determined by qPCR. Student’s *t*-test was used for statistical analysis of uptake data.

## Results

Histamine uptake in neonatal rat astrocytes is a bidirectional process, which was found to be dependent on pH and Na^+^, but not Cl^−^-dependent, with low affinity (K_m_ 116 µM) and high capacity (158 pmol/mg protein). The uptake was inhibited by millimolar concentrations of other biogenic amines (dopamine, noradrenaline and 5-hydroxytryptamine). The histamine metabolite tele-methylhistamine affected both directions of histamine uptake. In spite of the presence of OCT2 in neonatal astrocytes, the OCT inhibitors decynium-22 and corticosterone had no affect on histamine clearance.

## Conclusions

Histamine is taken up into astrocytes by low-affinity high-capacity uptake, which involves transporter(s) other than OCT.

